# Fluoropyrimidine Modulation of the Anti-Tumor Immune Response―Prospects for Improved Colorectal Cancer Treatment

**DOI:** 10.3390/cancers12061641

**Published:** 2020-06-21

**Authors:** William H. Gmeiner

**Affiliations:** Department of Cancer Biology, Wake Forest School of Medicine, Winston-Salem, NC 27157, USA; bgmeiner@wakehealth.edu

**Keywords:** 5-Fluorouracil, thymidylate synthase, immunogenic cell death, immunotherapy, MDSCs, T-cells

## Abstract

Chemotherapy modulates the anti-tumor immune response and outcomes depend on the balance of favorable and unfavorable effects of drugs on anti-tumor immunity. 5-Florouracil (5-FU) is widely used in adjuvant chemotherapy regimens to treat colorectal cancer (CRC) and provides a survival benefit. However, survival remains poor for CRC patients with advanced and metastatic disease and immune checkpoint blockade therapy benefits only a sub-set of CRC patients. Here we discuss the effects of 5-FU-based chemotherapy regimens to the anti-tumor immune response. We consider how different aspects of 5-FU’s multi-factorial mechanism differentially affect malignant and immune cell populations. We summarize recent studies with polymeric fluoropyrimidines (e.g., F10, CF10) that enhance DNA-directed effects and discuss how such approaches may be used to enhance the anti-tumor immune response and improve outcomes.

## 1. Introduction

Immune surveillance is essential for limiting cancer incidence and an effective anti-tumor immune response is important for maintaining durable remissions in colorectal cancer (CRC) patients with locally advanced and metastatic disease. The most widely used drug for CRC treatment is 5-Fluorouracil (5-FU) [1,2,3]. Biochemical and immunomodulatory approaches to enhance 5-FU’s efficacy have been implemented for decades [4]. For example, 5-FU+levamisole conferred a survival benefit for CRC patients with locally advanced disease (Duke’s stage C) [5]. Levamisole is an immune modulating agent [6] that displayed synergy with 5-FU in CRC cells by increasing expression of class I human leukocyte antigens (HLA-1) [7]. Both type I and II interferons also were evaluated in combination with 5-FU and strong efficacy was found in some clinical studies consistent with their immunomodulatory properties [8]. Ultimately, immunomodulatory approaches to enhancing 5-FU efficacy were shown to be inferior to biochemical modulation with leucovorin (LV) [9], a reduced folate [10] that promotes thymidylate synthase (TS) inhibition by 5-FU [11]. TS is essential for de novo pyrimidine biosynthesis and its inhibition causes malignant cells to undergo “thymineless death” [12], a well-validated strategy for cancer treatment [3]. In recent years, there is increasing emphasis on enhancing the anti-tumor immune response thru use of immune-checkpoint blockade (ICB) [13]. While this strategy has impacted outcomes considerably in non-small cell lung cancer (NSCLC) [14], melanoma [15] and other malignancies [16], it has a lesser impact in CRC [17]. Understanding how 5-FU chemotherapy regimens affect the anti-tumor immune response in CRC is critical to devising new ways to harness the immune system to improve outcomes for CRC patients.

In this review, we discuss the survival benefit associated with adjuvant chemotherapy with 5-FU-based regimens for CRC [18] in the context of therapy-induced effects not only to malignant cells but also to the host anti-tumor response. We first summarize evidence for the survival benefit associated with 5-FU-based regimens and describe 5-FU’s multi-factorial cytotoxic mechanism [19] that includes both DNA-directed [20,21] and RNA-mediated effects [22,23,24], as well as potential toxic effects resulting from degradation metabolites. We then summarize evidence that 5-FU modulates the anti-tumor immune response by reducing immunosuppressive cell populations (myeloid-derived suppressor cells (MDSCs), Tregs) and by stimulating immunogenic cell death (ICD,–i.e., damaging malignant cells to recruit their phagocytosis by dendritic cells (DCs) and ultimately generating an anti-tumor response mediated by effector T-cells. The favorable immunomodulatory properties of 5-FU are countered, however, by immunosuppressive and pro-inflammatory effects. Interestingly, both 5-FU-induced lymphodepletion [25] and gastrointestinal (GI)-tract inflammation [23,26] result from 5-FU’s RNA-mediated effects. We summarize recent studies from our laboratory [27] that demonstrate that polymeric fluoropyrimidines (FPs; for example, F10, CF10 [28]) with primarily DNA-directed cytotoxic mechanism display improved anti-tumor activity and reduced GI-tract and hematopoietic toxicities relative to 5-FU. Thus, it may be possible to harness the anti-tumor immune response more effectively by altering the metabolite distribution of FPs to be more DNA-directed.

## 2. Results

Colorectal cancer (CRC) is a major cause of cancer-related mortality and causes 700,000 deaths annually worldwide [29] (51,000 in the U.S.). The mortality associated with colon cancer results almost exclusively from metastatic disease. Five-year survival rates for patients diagnosed at the regional and distant stages are 71% and 13%, respectively [30]. Most patients with limited-stage disease undergo potentially curative surgical resection and adjuvant chemotherapy is administered to patients with stage III and high-risk stage II disease to eradicate micrometastatic disease. A survival benefit for adjuvant chemotherapy with 5-fluorouracil (5-FU)-based regimens in stage III CRC was first demonstrated in 1988 in the NSABP C-01 trial [31]. 5-FU-based regimens have been used since to reduce risk for disease recurrence and improve overall- and disease-free survival. 5-FU in combination with the reduced folate leucovorin (LV) has a demonstrated survival benefit in the adjuvant setting for stage II and III CRC [32], as well as a survival benefit for stage IV disease. 5-FU-based chemotherapy regimens such as FOLFOX (5-FU/LV/Oxaliplatin) [33] and FOLFIRI (5-FU/LV/Irinotecan) [34] display a further improved survival benefit in the metastatic setting relative to 5-FU/LV and these regimens are now in widespread use.

There is a growing appreciation that the survival benefit derived from 5-FU-based chemotherapy regimens is multi-factorial, resulting not only from direct cytotoxic effects to cancer cells but also by modulating the host anti-tumor immune response [35]. 5-FU modulates the host anti-tumor response by affecting multiple cell types. For example, 5-FU may cause some tumor cells to be more visible to the adaptive immune system, resulting in enhanced eradication of tumor cells by effector T-cells [36]. Further, 5-FU may be cytotoxic to immunosuppressive MDSCs [37], restoring anti-tumor immunity. However, any favorable impact of 5-FU to anti-tumor immunity may be countered by processes that attenuate any potential anti-tumor immune response. 5-FU causes GI-tract inflammation in most patients [38], resulting in chronic inflammation that may attenuate anti-tumor immunity. Further, 5-FU is myeloablative [39], with neutropenia and leukopenia occurring in many patients [25,40].

### 2.1. Cytotoxic Mechanism of 5-FU 

Mechanistically, 5-FU is complex. 5-FU enters malignant cells by facilitated diffusion [41] and competes with Ura for metabolism by enzymes that mediate de novo pyrimidine biosynthesis [42]. 5-FU-derived metabolites affect both RNA- and DNA-mediated processes [42]. While most 5-FU-derived metabolites are processed with a similar efficiency to that of the native substrates, three types of metabolites display altered biochemical properties and are deleterious to specific cell types [19]—(i) DNA-directed (e.g., FdUMP, FdUTP); (ii) RNA-directed (FUTP); and (iii) degradation products (e.g., FBAL). Most 5-FU administered to humans (~85%) is degraded or excreted intact [43] and patients deficient in 5-FU catabolism due to genomic polymorphisms present in 5–10% of the population are at risk for serious and potentially lethal 5-FU toxicities [44,45] that may require therapeutic monitoring [46]. Among anabolic metabolites, ribonucleotides predominate over deoxyribonucleotides [47] and are associated with systemic toxicities that are reversed with Uridine [48], while DNA-directed effects are primarily responsible for the anti-tumor response [3].

#### 2.1.1. DNA-Directed Effects of 5-FU 

**Figure 1:**
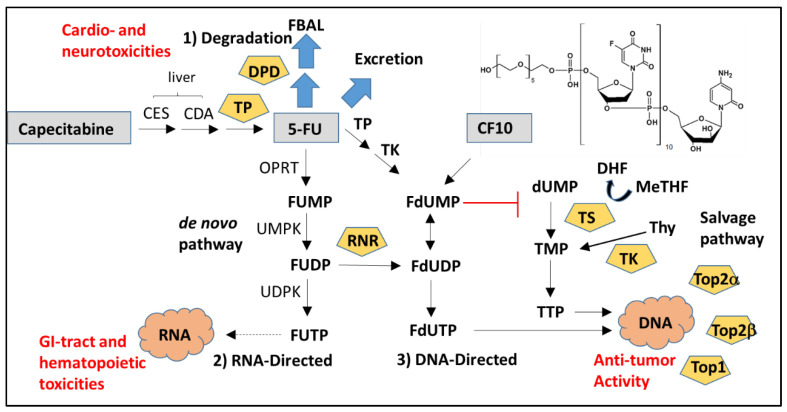
Overview of fluoropyrimidine metabolism. Fluoropyrimidines exert biological effects thru three types of metabolites: (**1**) Degradation; (**2**) RNA-directed; and (**3**) DNA-directed. Most 5-FU is either degraded or excreted intact and degradation metabolites contribute to cardio- and neurotoxicities. RNA-directed metabolites cause GI-tract and hematopoietic toxicities and contribute to immunosuppression. DNA-directed metabolites are responsible for the anti-cancer activity of fluoropyrimidines. Fluoropyrimidine polymers (e.g., CF10) are more efficiently converted to DNA-directed metabolites and may reduce the immunosuppressive and pro-inflammatory effects of 5-FU.

While 5-FU is inefficiently converted to deoxyribonucleotide metabolites (<5% of administered dose) thru a multi-step process (Figure 1) [47], these metabolites are primarily responsible for 5-FU’s direct cytotoxic effects to malignant cells [3]. FdUMP is a potent inhibitor of thymidylate synthase (TS), which catalyzes the reductive methylation of dUMP to thymidylate (TMP) [11]. TS is required for de novo pyrimidine biosynthesis required to support rapid proliferation of malignant cells. TS inhibition causes an imbalance in deoxynucleotide pools (i.e., elevated dATP/TTP ratio [49]) that contributes to replication stress [50]. Further, under the ensuing thymineless conditions, FdUTP becomes misincorporated into genomic DNA, which causes the trapping of DNA topoisomerase 1 cleavage complexes (Top1cc) [51,52]. Top1cc formation exacerbates replication stress and causes potentially lethal DNA double strand breaks due to collision with advancing replication forks [51]. We recently reviewed the entrapment of DNA topoisomerase-DNA complexes by nucleoside analogs and its role in cancer cell death [21]. Thymineless death induced by FPs involves activation of the extrinsic apoptotic pathway, which occurs via upregulation of Fas/FasL expression [53,54] or by altered sub-cellular localization of Fas death receptor without increased Fas expression [55] and sensitizes malignant cells to agonistic anti-Fas antibodies [55], consistent with sensitization to T-cell-mediated killing [56].

#### 2.1.2. RNA-Directed Effects of 5-FU

While rapidly proliferating malignant cells undergo thymineless death in response to 5-FU treatment [3], non-malignant cells, both differentiated and proliferative, undergo primarily apoptotic cell death due to altered RNA processing induced by 5-FU [23]. We and others, have shown that 5-FU incorporation into RNA perturbs RNA structure [57,58,59] and stability [60] contributing to its altered function [24]. 5-FU causes GI-tract toxicities that may be serious and are occasionally lethal, particularly in cancer patients deficient in 5-FU catabolism due to polymorphisms in *DPYD* [43]. 5-FU-induced GI-tract toxicities are reversed by Uridine (Urd) [48], consistent with an RNA-mediated process. 5-FU also causes myelosuppression [40], which may increase risk for infection [61]. 5-FU induced leukopenia may be reversed by Urd [25], consistent with an RNA-mediated origin. The effects of 5-FU on hematopoiesis [39] and on mature hematopoietic cells are important for understanding 5-FU’s overall modulation of the immune anti-tumor response. 

#### 2.1.3. Effects of 5-FU Degradation Metabolites

While patients deficient in 5-FU catabolism are at increased risk for 5-FU toxicity mainly from elevated levels of ribonucleotide metabolites, the products of 5-FU catabolism, (α-fluoro-β-alanine (FBAL) [62] and fluoroacetate [63]), cause cardio [64]- and neurotoxicities [65] and are associated with hyperammonemia [66] that may be lethal. FBAL is an amino acid analog and its toxic effects may result from misincorporation into proteins while fluoroacetate could disrupt the tricarboxylic acid cycle [66]. 5-FU’s degradation products have not been reported to affect immune cell function or the anti-tumor immune response; however, T-cell metabolism is important for anti-tumor immunity [67] and non-native metabolites including FBAL and fluoroacetate could exert a disruptive effect.

## 3. 5-FU Modulates the Anti-Tumor Immune Response

Most, if not all, chemotherapy drugs affect the anti-tumor immune response to some extent [68]. The extent of modulation likely depends on the drug, the tumor-type and stage and the genomic characteristics of individual tumors, among a multitude of factors. The potential for chemotherapy to favorably impact the anti-tumor immune response may be considered in terms of two categories [69]—(i) Attenuating Immunosuppressive Cell Populations; and (ii) Stimulating Immunogenic Cell Death (ICD). In the first category, chemotherapy selectively eradicates cell populations that suppress the anti-tumor immune response (e.g., Treg, MDSC). In the second category, chemotherapy induces tumor cell death in a manner that renders dying tumor cells more visible to the immune system. These categories are not exclusive and an anti-cancer drug may modulate anti-tumor immunity thru processes in both categories as summarized for 5-FU in Section 3.1 and Section 3.2, respectively. However, 5-FU also activates processes that are disruptive to the anti-tumor immune response that counter potentially favorable effects to anti-tumor immunity. 5-FU damages cells in the GI-tract [23] and this damage initiates an inflammatory response that is mediated thru IL-4 [26], a cytokine upregulated in many colon cancer patients that may adversely affect the anti-tumor immune response [70]. Further, 5-FU alters the composition of the gut microbiome, which also affects the anti-tumor immune response [71].

### 3.1. 5-FU Effects to MDSCs and T_Regs_

Myeloid-derived suppressor cells (MDSCs) are heterogeneous immature myeloid cells that fail to terminally differentiate and suppress the anti-tumor activities of T and NK cells [72]. In response to acute inflammation, MDSCs expand and differentiate into monocytes and neutrophils in a process knowns as myelopoiesis [73]. In cancer, MDSCs expand and become activated but they do not fully differentiate into monocytes and neutrophils. MDSCs accumulate in tumor and peripheral lymphoid organs in tumor-bearing hosts and impact effector cell function thru multiple mechanisms that include [74]—(i) inhibiting CD4+ and CD8+ T-cell proliferation and activation; (ii) altering macrophage to a type 2 phenotype; (iii) inhibiting the cytotoxicity of NK cells; and (iv) inducing Treg cells to escalate immunosuppression. 

**Figure 2:**
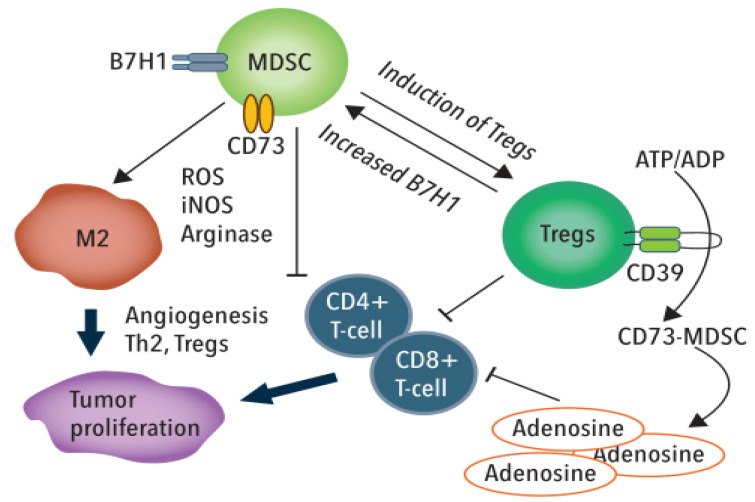
Myeloid-derived suppressor cells (MDSCs) and T_regs_ suppress the anti-tumor activity of T-cells thru multiple mechanisms.

Tregs are a sub-population of CD4+ T-cells that display immunosuppressive function. Specifically, TRegs suppress conventional T helper (Th) cells and contribute to maintenance of immunologic self-tolerance [75]. TRegs infiltrate into the tumor microenvironment attracted by chemokine gradients (e.g., CCR4-CCL17/22) and, upon activation, inhibit antitumor immune responses. Effector/activated Treg cells (eTreg) inhibit maturation of antigen-specific DCs and also exert non-specific immunosuppressive effects through IL-2 consumption and degradation of ATP to adenosine which impairs T-cell function [76,77] (Figure 2)., Further, Tregs secrete immunosuppressive cytokines IL-10, TGF-β and Il-35 [78] and undergo proliferation in response to tumor-derived factors including TGF-β and IL-10 [79]. eTReg also express immune checkpoint molecules (e.g., CTLA-4) to inhibit cytotoxic T-cells and suppress the anti-tumor immune response [80].

Clinical studies show increased MDSC (CD33+CD11b+HLA−DR−) are present in tumor tissue relative to para-neoplastic tissue [81]. Further, the MDSC percentage in PBMC from CRC patients was significantly greater than from healthy donors and both MDSC and Treg (CD4+CD25highFOXP3+) populations in PBMCs significantly decreased following tumor resection. CRC cells promote MDSC expansion, which suppresses T cell proliferation resulting in enhanced CRC cell growth. The clinical significance of MDSC levels for CRC outcomes was demonstrated by studies showing elevated CD33+ MDSC cells in CRC patients were associated with significantly reduced disease-free and overall survival [82]. Mechanistic studies revealed tumor YAP1 expression promoted MDSC induction by stimulating granulocyte-macrophage colony-stimulating factor (GM-CSF) secretion thru increased COX-2, pAkt and P-p65. While Tregs are increased in CRC, effects are complex and Tregs may have protective or suppressive function depending on disease stage among other factors [83,84].

Among the chemotherapeutic drugs shown to selectively deplete MDSCs in pre-clinical studies are Gemcitabine (Gem) [85] and 5-FU [37]. In a 4T1/Balb-c breast cancer syngeneic model Gem but not cyclophosphamide, significantly decreased both %-MDSC in the spleen and absolute MDSC number. While MDSC depletion rescued T-cell function, it did not enhance anti-tumor activity. 5-FU treatment in a syngeneic thyoma model was superior to Gem in MDSC depletion through increased induction of MDSC apoptosis. 5-FU induced MDSC depletion, promoted IFNγ production by tumor-infiltrating CD8+ T-cells and stimulated a T-cell-dependent antitumor effect. While 5-FU induced MDSC depletion in some tumor models, 5-FU’s effects on MDSCs is dependent both on the tumor model and the dosing regimen [74]. MDSC numbers were not significantly decreased 7 days post-treatment in studies in which 5-FU was dosed repeatedly, indicating MDSC number may stabilize with repeated treatment. Further, 5-FU activates the inflammasome in dying MDSCs leading to IL-1β secretion and IL-17 production by Th17 cells that increased angiogenesis and stimulated tumor growth. 5-FU also modulates levels of TRegs in a dose- and time-dependent manner [86]. 

Clinical studies demonstrated that 5-FU-based regimens modulate levels of immunosuppressive cells and that a favorable response is associated with chemotherapy-induced reduction in immunosuppressive cell populations [87,88]. Elevated levels of granulocytic MDSCs (gMDSCs) were associated with poor prognosis in a longitudinal study of mCRC patients treated with FOLFOX-bevacizumab. Patients in which FOLFOX-bevacizumab treatment decreased gMDSC levels displayed a better survival outcome than those that did not, although these studies do not distinguish direct cytotoxic effects to immunosuppressive cell populations from indirect effects. FOLFOX-bevacizumab therapy was also associated with decreased Treg and increased Th17 cell frequency [87]. However, 5-FU-based regimens are not uniformly effective at reducing immunosuppressive cell populations as FOLFIRI displayed an opposite effect from FOLFOX on MDSC populations [88]. Effects of 5-FU to TReg levels and influence on outcomes is not as defined as for MDSCs although the CD8:Treg ratio is associated with favorable outcomes in mCRC patients treated with anti-VEGF therapy.

MDSCs and Tregs are important mediators of immunosuppression affected by chemotherapy [89] but other cell populations that contribute to immunosuppression may be responsive to 5-FU chemotherapy. Upregulation of cytotoxic T-cell cell populations specific for tumor antigens is central to an anti-tumor immune response and elevated CD3+ and CD8+ tumor-infiltrating lymphocytes correlates with a favorable outcome in CRC [90,91]. Clinical studies indicate select leukocyte sub-populations detected in peripheral blood mononuclear cells (PBMCs) systematically differ between CRC patients and healthy controls. Consistent with CRC patients presenting with a general immunocompromised state, CRC patients and healthy donors displayed similar proportions of circulating T-cells (both CD4+ and CD8+), NK cells and NKT cells [92] but circulating T-regs were increased in CRC patients [79]. While the total NK population in PBMCs did not differ between CRC patients and healthy donors, CRC patients displayed reduced expression of natural cytotoxicity receptors (NCRs) NKp44 and NKp46 on CD56dim NK cells and NKT-like cells. NCRs mediate NK cell killing and IFNγ release [93]. The effects of NK levels on CRC outcomes is controversial with one study reporting above-median percentage of CD16+ NKT-like cells was associated with decreased disease-free survival for CRC patients [92], while another study found the percentage of NK cells in blood was an independent predictor of survival in CRC patients [94]. CD16 activates resting NK cells thru engagement with antibodies; however, NK cells may become exhausted and show lower cytotoxic activity thru CD16 stimulation [95]. CD16+CD56+ NK cells post-chemotherapy with 5-FU-based regimens also negatively correlated with outcomes consistent with chemotherapy potentially modulating outcomes by affecting specific sub-sets of NK cells [96]. In this regard, we have shown that 5-FU decreases viability of an immortalized NK population ex vivo, while the DNA-directed fluoropyrimidine (FP) polymer CF10 does not (Gmeiner and Soto-Pantojo, in preparation). Reduction of CD16+ NK cells following 5-FU-based chemotherapy was also detected in clinical studies [97].

### 3.2. 5-FU Stimulation of Immunogenic Cell Death

Malignant cells are induced by chemotherapy to undergo any one of several cell death processes (e.g., apoptosis, necrosis, etc.). The mode of cell death is an important determinant in activating immunogenic cell death (ICD), an immune response capable of contributing to further tumor eradication. In general, the extent to which chemotherapy-induced malignant cell death is immunogenic depends on both the antigenicity of target malignant cells (i.e., expression of cancer-specific epitopes) and adjuvanticity or propensity to enhance cross-presentation of cancer-specific antigens to CD8+ T-cells by dendritic cells via MHC-I. Antigenicity is determined, in part, by mutational burden, which depends on DNA mismatch repair among other factors. Microsatellite instable (MSI) CRC tumors, in general, having greater mutational burden than MSS disease. Recruitment of DCs to dying cancer cells is stimulated by the secretion of damage-associated molecular patterns (DAMPS) [98]. A necessary step in the recognition of dying malignant cells by phagocytes is Calreticulin (CRT) cell surface expression. CRT is an endoplasmic reticulum (ER) chaperone and its presentation in complex with ERp57 [99] on the surface of malignant cells provides a potent “eat me” signal for phagocytic engulfment via the HSP protein-CD91 pathway [100]. DCs and macrophages localize to dying cells via an ATP gradient (“find me” signal), created by autophagy-dependent ATP release from dying cells [101]. The licensing of DCs to process and present tumor antigens requires interaction of DAMPs (e.g., HMGB1, HSP70) released from dying tumor cells, with Toll-like receptor 4 (TLR4) on DCs [102]. The relevance of this process for the immunogenicity of cancer chemotherapy is demonstrated by a TLR4 polymorphism affecting HMGB1 binding predicting relapse in breast cancer patients treated with anthracycline chemotherapy [102]. DCs that phagocytose dying tumor cells increase presentation of tumor-associated antigens and elicit cytotoxic responses by autologous lymphocytes.

Chemotherapeutic drugs may be classified as ICD-inducers, in part, based on vaccination assays in which mice injected with drug-treated tumor cells are protected against subsequent re-challenge with the same tumor. Using this and related criteria several chemotherapy drugs have been categorized as ICD-inducers including anthracyclines, cyclophosphamide, mitoxantrone and bortezomib [103]. Recent studies indicate that by the same criteria, 5-FU and clinically relevant combinations are ICD-inducers [36]. 5-FU in combination with oxaliplatin and leucovorin comprises the FOLFOX regimen widely used for CRC treatment. Both 5-FU and OXA induced HMGB1 and HSP70 release from CRC cells and the 5-FU/OXA combination was more effective than either drug at inducing DAMP secretion. Further, HMGB1 and HSP70 were increased in serum from CRC patients following treatment with FOLFOX establishing potential clinical relevance [36]. Supernatants from 5-FU and 5-FU/OXA-treated human CRC cells induced maturation of human DCs based on upregulated HLA-DR, CD80 and CD86 and DC maturation was inhibited by antibodies to TLR4. Analogous effects were detected with mouse CRC cells and mouse DCs. Finally, DCs pulsed with supernatants from 5-FU and 5-FU/OXA-treated mouse cancer cells displayed an enhanced anti-tumor effect relative to immunization with DCs that were pulsed with supernatants from untreated cells. While these studies are supportive of a role for ICD in contributing to the anti-tumor effects and survival benefit of 5-FU-based chemotherapy regimens in CRC, these studies used a single syngeneic model (CT26/Balb-c) that may not be reflective of human CRC. In particular, CT-26 expresses wild-type p53 [104], while approximately half of CRC tumors express mutant p53 [105]. This is significant because the response of CRC cells and induction of apoptosis following 5-FU-treatment depends on p53 expression [106]. Further, the efficacy of adjuvant 5-FU in stage III colon cancer patients is limited to patients expressing wt-p53 [107]. In this regard, we are developing polymeric FPs that differ from 5-FU and other anti-cancer drugs and these induce apoptosis regardless of p53 expression [108]. Further studies are needed to establish the generality and limitations of 5-FU-induced ICD in murine model systems and to develop FPs that are more potent and more general inducers of ICD in CRC patients.

### 3.3. 5-FU Effects on Immune Cells Are Dynamic

5-FU chemotherapy is immunosuppressive and a linear relationship between 5-FU plasma concentration and decreased leukocyte count was observed [109]. A recent study indicated 70% of CRC patients treated with 5-FU according to the Mayo schedule experienced ≥grade 1 hematological toxicities (neutropenia and/or leukopenia) [40]. Further, 5-FU toxicity increases healthcare costs with toxicity-related hospitalizations occurring at a higher rate in 5-FU treated patients relative to those not receiving chemotherapy (31% vs. 8%) with increased cost of $2716 per patient [110]. While 5-FU causes immunosuppression in many patients, studies in mice reveal the effects of 5-FU on hematopoietic populations are dynamic. Thus, 5-FU′s initial myeloablative effects stimulate a rebound response that tends to restore steady-state levels [39], which is consistent with 5-FU′s net impact on anti-tumor immunity reflecting a balance between opposing forces. 5-FU treatment resulted in a rebound in HSCs following initial reduction. HSC increase was Tpo-regulated and 5-FU but not irradiation, induced overexpression of Tie-2/Angpt-1 by stromal cells of the bone marrow [39]. 5-FU eliminates committed progenitors in bone marrow but also effects long-term reconstituting stem cells by decreasing expression of c-kit [111]. While a rebound effect in HSCs following single, high dose 5-FU treatment was observed in mice [39], the effects of multiple treatments simulating clinical regimens are more complex. Studies using the CT26/Balb-c model demonstrated that multiple cycles of 5-FU treatment improved tumor growth inhibition better than a single cycle but more extensive dosing did not improve survival [112]. Immune cell sub-populations from PBMCs did not differ between treated and control groups except a significant reduction in B-cells with multiple treatments. Multiple cycles of 5-FU, however, decreased proliferation of CD8+ T-cells specific for CT26, indicating the potential for 5-FU to attenuate anti-tumor immunity in some instances by inhibiting proliferation of tumor-specific T-cell populations [112].

## 4. Modulation of 5-FU-Induced Anti-Tumor Immunity

### 4.1. Direct and Indirect Modulation of 5-FU by IFNs

Multiple studies have investigated the potential of interferons (IFNs) to enhance the anti-tumor activity of 5-FU in colorectal cancer patients. Several clinical studies evaluated 5-FU in combination with the type I interferon IFNα. Clinical studies were initiated in response to pre-clinical studies that demonstrated synergy for the IFNα/5-FU combination towards CRC cells [8,113]. Synergy was associated with increased thymidine phosphorylase expression [114], increased FdUMP levels, enhanced TS inhibition and greater DNA damage [115]. IFN-α2b was also found to modulate 5-FU pharmacokinetics [116], decreasing clearance, in part, by decreasing activity of dihydropyrimidine dehydrogenase [117]. IFNα could potentiate 5-FU activity for CRC treatment by enhancing activities of effector cell or modulating display of HLA class I antigens [118]. Meta-analysis of data from multiple trials concluded however that IFNα did not increase the efficacy of 5-FU or 5-FU+LV and that 5-FU+IFNα was significantly inferior to 5-FU+LV [9]. The type II IFN IFNγ was also evaluated for enhancing 5-FU’s anti-tumor activity. IFNγ is associated with anti-proliferative and anti-tumor mechanisms but also may have pro-tumor activities (downregulating MHC, upregulating PDL1) and clinical studies have had limited success [119]. Both 5-FU and IFNγ increased expression of carcinoembryonic antigen (CEA) by CRC cells although the combination was not synergistic. 5-FU and IFNγ both increased MHC I by CRC cells but neither induced expression of the co-stimulatory molecule B7-1 [120]. IFNγ did not enhance 5-FU-mediated DNA damage [115]. IFNγ did not display single agent activity in colon cancer [121], although promising results were obtained for the 5-FU/IFNγ combination for treatment of advanced hepatocellular carcinoma [122].

Indirect activation of interferon genes may also contribute to anti-tumor immunity thru activation of the STING (stimulator of interferon genes) pathway. STING activates interferon regulatory factor 3 (IRF3) and NF-κB stimulating production of cytokines and type I interferons. STING is activated in response to sensing of foreign DNA (i.e., viral, bacterial) by cyclic GMP-AMP synthase (cGAS). While the STING pathway primarily functions to sense foreign DNA and stimulate an immune response, STING also is activated in response to genomic DNA in the cytosol that could result from aberrant cell division or treatment-induced DNA damage. Radiation increases tumor production of IFNβ and antitumor efficacy of radiation depends on IFN signaling [123]. Further, IFNβ production in response to radiation of tumor cells is STING-dependent [124]. Thus, treatments that induce DNA damage, including fluoropyrimidine drugs, could potentially result in cytosolic DNA and STING pathway activation and contribute to anti-tumor immunity, in part, thru stimulating production of type I interferons. In this regard, Mus81-mediated DNA damage promoted cytosolic DNA accumulation in malignant cells contributing to STING activation and an enhanced anti-tumor immune response [125]. Mus81 is activated in response to replication stress induced by anti-cancer drugs [126]. Our studies with polymeric fluoropyrimidines demonstrate these compounds are more efficient inducers of replication stress relative to 5-FU [50], consistent with STING pathway activation being relatively more important for polymeric FPs than for 5-FU. Recent studies indicate the potential for STING pathway activation to potentiate 5-FU treatment. Exogenous administration of cGAMP, the cyclic nucleotide product resulting from cGAS activation, exerts single-agent anti-tumor activity [127]. Further, cGAMP enhanced the activity of 5-FU and reduced its toxicity. Hence, indirect production of IFNβ thru exogenous activation of the STING pathway may enhance 5-FU efficacy, although this approach has not yet been evaluated in clinical trials.

The mechanism by which STING activation enhances antitumor immunity involves DC activation and priming antitumor responses thru effector CD8+ T-cells. STING is predominantly expressed in DCs, macrophages, T-cells and epithelial cells [128]. STING-deficient DCs display an impaired ability to cross-prime CD8+ T cells following tumor cell irradiation [124]. Thus, engulfed tumor cells with damaged DNA may activate STING in DCs resulting in IFNβ production functioning in a paracrine or autocrine manner to enhance tumor antigen cross-presentation to T-cells [129,130]. The positive anti-tumor immune effects mediated by the STING pathway are countered, however, by pro-tumorigenic effects in certain contexts. STING activation can result in chronic inflammation contributing to tumor progression, in part by inducing indoleamine 2,3 dioxygenase (IDO) expression [131], which activates Tregs and suppresses effector and helper T-cells. Further, STING deficiency decreased MDSC and promoted tumor CD8+ tumor infiltration in the Lewis lung carcinoma model, consistent with an immunosuppressive function [132]. The potential enhancement of 5-FU and other anti-cancer drugs by STING activation still requires clinical validation.

### 4.2. Combining 5-FU-Based Chemotherapy with Immune Checkpoint Blockade

An anti-tumor immune response including recognition of malignant cells by effector T-cells is essential for a durable response to any cancer treatment. Even in cases where activated T-cells that recognize tumor-specific antigens are present, the anti-tumor effect may be muted by upregulation of immune checkpoint molecules on the surface of malignant cells, effector T-cells and other cell populations. For some malignancies, including NSCLC and melanoma, the upregulation of PD-L1 by tumor cells and PD-1 and CTLA-4 by effector T-cells leads to inhibitory interactions that attenuate tumor-directed T-cell cytotoxicity and antibodies directed at inhibiting these checkpoint interactions have had a profound impact on outcomes. Immune checkpoint inhibitors (ICIs), such as Nivolumab and Pembrolizumab, directed against PD-1, Atezolizumab and Durvalumab directed against PD-L1 and Ipilimumab targeting CTLA-4 and PD-1 display strong efficacy in NSCLC [133]. The activity of checkpoint inhibitors in CRC, however, is presently limited to a sub-set of CRC patients with high microsatellite instability (MSI-H) being the predominant stratifying factor. MSI-H CRC displays increased mutational burden relative to MSS disease and is associated with more favorable outcomes [134], consistent with increased anti-tumor immune response [135]. However, efficient tumor eradication by the immune system is countered by elevated PD-L1 expression by MSI-H CRC cells and elevated PD-1 and CTLA-4 by effector T-cells [136]. Clinical studies with Nivolumab and Pembrolizumab display promising activity for MSI CRC and other patients with high mutational burden and their use is recommended for patients with chemoresistant, MMR-deficient metastatic CRC [137]. 

Pre-clinical studies evaluating chemotherapy in combination with ICIs have shown that while favorable interactions can occur, effects depend on the tumor model, the type of chemotherapy and the ICI. Chemotherapy, including 5-FU [138], upregulated PD-L1 expression on CRC cells with the effects greatest for camptothecin [139]. The combination of capecitabine+oxaliplatin chemotherapy enhanced anti-PD1 ICI in an MC-38 CRC model [140]. While neither 5-FU nor OXA significantly enhanced anti-PD1 therapy in a CT-26/Balb-c model, FOLFOX/anti-PD1 therapy was highly effective resulting in long-term survival [141]. FOLFOX was shown to upregulate PD-L1 expression on tumor cells and to induce tumor infiltration by PD1+ CD8 T cells and this induction of adaptive immune resistance was countered by anti-PD1 therapy. FOLFOX induced PD-L1 tumor cell expression and stimulated CD8+ T cell infiltration in CRC patients indicating anti-PD1 therapy may be used effectively in combination with FOLFOX for CRC treatment [141]. The potential for ICI to enhance the efficacy of 5-FU-based chemotherapy is currently undergoing clinical investigation [142]. A Phase II study of FOLFOX and anti-PD-1 (Pembrolizumab) achieved an overall objective response rate of 53%, which was particularly encouraging in that the patient population was predominantly MSS CRC [143]. The combination was safe and tolerable with no grade 4-5 treatment related adverse effects [144]. While 5-FU in the context of FOLFOX appears promising for enhancing anti-PD1/PD-L1 ICI therapy and extending ICI to MSS disease, the mechanistic basis is not known. In particular, 5-FU the relative contribution of TS inhibition and DNA-directed effects relative to RNA-mediated effects requires further elucidation

## 5. Novel Chemical Approaches to Modulating 5-FU’s Anti-Tumor Response

TS inhibition [11] is central to the anti-tumor activities of 5-FU [3] and may be important for stimulating the anti-tumor immune response and enhancing activity of ICI therapy. However, a major limitation of 5-FU is its inefficient conversion to FdUMP [47]. In principle, the deoxynucleotide FdU would generate reduced ribonucleotide metabolites relative to 5-FU; however, FdU is rapidly taken up by the liver and metabolized to 5-FU and its clinical use is limited to hepatic arterial infusion for treatment of liver metastasis [145,146]. 5-FU is now always co-administered with LV to enhance TS inhibition under low folate conditions; however, this does not reduce toxicities nor does it improve overall survival [19]. To overcome this limitation we have developed polymeric FPs (e.g., F10, CF10) that are more directly converted to FdUMP [27] and display greater TS inhibitory activity, cause greater DNA damage and exert improved anti-tumor activity relative to 5-FU in multiple pre-clinical models [108,147,148,149]. 

We recently demonstrated the 2nd generation polymeric FP CF10 displayed significantly improved anti-tumor activity relative to 5-FU in an orthotopic colon cancer model consistent with the potential of polymeric FPs to provide a survival benefit for CRC patients. The realization of a clinical benefit for CF10 depends on multiple factors among which effects on anti-tumor immunity are of high importance. CF10 is expected to differ from 5-FU on its effects to anti-tumor immunity because mechanistically it is more DNA-directed while 5-FU exerts both RNA- and DNA-directed effects. The extent of the mechanistic difference between F10 and 5-FU was demonstrated by COMPARE analysis of data from the NCI60 cell line screen, which showed low correlation between these drugs consistent with dissimilar mechanisms [52]. Further, we showed 5-FU-induced apoptosis in CRC cells was p53-dependent and rescued by Uridine while F10’s effects were p53-indepedendent and not reversed by Uridine [150]. Apart from distinguishing polymeric FPs from 5-FU mechanistically, these findings have potential implications for anti-tumor immunity since mode of cell death affects DAMP secretion, DC cell maturation and ultimately the recognition of malignant cells as foreign by effector T-cells. Additional studies are needed to determine if polymeric FPs differ from 5-FU in these important endpoints. CF10 and F10 display improved anti-tumor activity relative to 5-FU in both syngeneic tumor models [108,149] and xenograft studies in immunodeficient mice consistent with polymeric FPs not inducing decreased levels of immunogenic cell death relative to 5-FU.

The RNA-mediated effects of 5-FU cause GI-tract [23] and hematopoietic toxicities [25], both of which attenuate the anti-tumor response. Consistent with its mechanism being primarily DNA-directed, we have demonstrated that CF10 induces less GI-tract damage and less hematopoietic toxicity than 5-FU. 5-FU-induced GI-tract inflammation is IL-4-dependent [26] and IL-4 is upregulated in CRC and contributes to an immunosuppressive environment [70]. Since CF10 causes reduced GI-tract damage and less inflammation than 5-FU, it may exert reduced immunosuppressive effects contributing to an improved overall anti-tumor response. GI-tract damage and inflammation result from RNA-mediated processes perturbed by 5-FU that have not been elucidated in molecular detail. While both GI-tract and hematopoietic tissues include proliferative cells that are potentially vulnerable to CF10’s DNA-directed activities, proliferation in non-malignant tissue may use the salvage pathway for Thy needed for DNA replication, while malignant cells may be more reliant on de novo Thy biosynthesis. This difference on reliance on de novo Thy biosynthesis may provide the basis for the relatively larger therapeutic window we observe for CF10 relative to 5-FU in murine anti-tumor studies. Ultimately, clinical studies are needed to determine if the therapeutic advantages for CF10 relative to 5-FU also occur in humans and if CF10 initiates a more favorable anti-tumor immune response and improves outcomes. 

## 6. Conclusions

5-FU and 5-FU-based regimens display a survival benefit in stage II, III and IV CRC. The effects of 5-FU to the anti-tumor immune response are an important consideration both in understanding efficacy achieved with 5-FU and in developing improved FP drugs and novel combinations that might improve survival, which remains dismal for CRC patients with late-stage disease. 5-FU exerts biological effects thru DNA-, RNA- and degradation metabolites [19]. The anti-tumor activity of 5-FU results primarily from TS inhibition and DNA-directed metabolites but 5-FU is inefficiently converted to deoxynucleotide metabolites [47]. The RNA-directed activities of 5-FU cause GI-tract [23] and hematopoietic [25] toxicities that may be serious, particularly in patients deficient in 5-FU catabolism [43]. While in most instances these toxicities are manageable, they are occasionally lethal. Further, lymphodepletion and GI-tract inflammation may contribute to 5-FU-induced immunosuppression that limits the anti-tumor immune response contributing to sub-optimal outcomes. While some studies reported that 5-FU decreased immunosuppressive MDSC and Treg cell populations consistent with enhancing anti-tumor immunity, 5-FU also was shown to activate the inflammasome in dying MDSCs leading to IL-1β secretion that increased angiogenesis and stimulated tumor growth. 5-FU also induced DAMP secretion from dying CRC cells that activated DCs, which could then be used to stimulate a T-cell-mediated response to the same tumor type in other mice. The immunogenic effects of 5-FU may be limited to malignancies with a restricted genomic profile, such as wtp53, that is required for it to efficiently activate apoptosis. The polymeric FP CF10 induced primarily DNA-directed cell death processes and greater anti-tumor activity than 5-FU in multiple pre-clinical models. CF10 may be effective at inducing immunogenic cell death in malignancies in which 5-FU is ineffective. CF10 and F10 also caused less GI-tract inflammation and less hematopoietic toxicity than 5-FU [108], consistent with reduced immunosuppression. The effects of CF10 on modulating immunogenic cell death warrant further investigation.
